# Preparation of Hydrogel Composites Using a Sustainable Approach for In Situ Silver Nanoparticles Formation

**DOI:** 10.3390/ma16062134

**Published:** 2023-03-07

**Authors:** Laura Chronopoulou, Roya Binaymotlagh, Sara Cerra, Farid Hajareh Haghighi, Enea Gino Di Domenico, Francesca Sivori, Ilaria Fratoddi, Silvano Mignardi, Cleofe Palocci

**Affiliations:** 1 Department of Chemistry, Sapienza University of Rome, P. le A. Moro 5, 00185 Rome, Italy; 2 Research Center for Applied Sciences to the Safeguard of Environment and Cultural Heritage (CIABC), Sapienza University of Rome, P. le A. Moro 5, 00185 Rome, Italy; 3 Department of Biology and Biotechnology “C. Darwin”, Sapienza University of Rome, P. le A. Moro 5, 00185 Rome, Italy; 4 Microbiology and Virology, San Gallicano Dermatological Institute, IRCCS, Via E. Chianesi 53, 00144 Rome, Italy; 5 Department of Earth Sciences, Sapienza University of Rome, P. le A. Moro 5, 00185 Rome, Italy

**Keywords:** peptide hydrogel, composite, silver nanoparticles

## Abstract

The recognized antibacterial properties of silver nanoparticles (AgNPs) characterize them as attractive nanomaterials for developing new bioactive materials less prone to the development of antibiotic resistance. In this work, we developed new composites based on self-assembling Fmoc-Phe3 peptide hydrogels impregnated with in situ prepared AgNPs. Different methodologies, from traditional to innovative and eco-sustainable, were compared. The obtained composites were characterized from a hydrodynamic, structural, and morphological point of view, using different techniques such as DLS, SEM, and rheological measurements to evaluate how the choice of the reducing agent determines the characteristics of AgNPs and how their presence within the hydrogel affects their structure and properties. Moreover, the antibacterial properties of these composites were tested against *S. aureus*, a major human pathogen responsible for a wide range of clinical infections. Results demonstrated that the hydrogel composites containing AgNPs (hgel@AgNPs) could represent promising biomaterials for treating *S. aureus*-related infections.

## 1. Introduction

Currently, one of the most interesting objectives from a scientific and biomedical point of view is the search for novel tools to fight bacterial infections that do not involve the use of antibiotics [1,2]. In fact, more and more often, clinicians face pathogenic microorganisms that have developed resistance against currently available antibiotics for treating bacterial infections. Therefore, developing alternative antibiotic agents and treatments to control bacterial infections is critical. A possible answer to this growing issue is the use of nanomaterials with intrinsic antimicrobial properties [3,4,5,6]. Silver nanoparticles (AgNPs) are among the most promising nanostructured materials in the biomedical field thanks to the antibacterial properties of silver. In fact, AgNPs are more reactive and therefore have a greater antibacterial activity as there is an increase in the available surface exposed to microbes (high surface/volume ratio typical of nanomaterials) [7,8,9]. A number of approaches are available for the synthesis of AgNPs, such as physical, electrochemical, and biological methods [10,11]. The role of the functionalizing layer (e.g., polymeric ligands, surfactants, thiols) has also been deeply investigated for surface protection and colloidal stability improvement, also in view of different applications [8,12]. The major problems that could be encountered using traditional methods for the preparation of AgNPs are related to the high cost and use of toxic chemicals that can represent a possible biological and environmental risk. Furthermore, AgNPs used in the biomedical field must meet precise standards of final purity. To overcome these problems, innovative biological synthesis methods, which are more sustainable and less harmful to both humans and the environment, have recently evolved [13,14,15]. These methods, compared to the chemical-physical ones, also allow a simpler control of the size, polydispersion, and shape of the obtained AgNPs. For example, some of the most widely used methods involve bacteria, fungi, and plant extracts [16,17,18].

Another approach being developed in the synthesis of AgNPs involves replacing classical chemical reducing agents, such as NaBH_4_, with carbohydrates [19]. Among these, the sugars with a greater reducing activity are monosaccharides. For a sugar molecule to have a reducing activity, it must exist in an open chain form with an aldehyde or ketone group, in the case of an aldose or a ketose, respectively. The reducing function is performed by the carbonyl group, which transfers electrons to another species, e.g., to Ag^+^, producing Ag° clusters [20]. Several works in the literature report the reduction of silver salts using carbohydrates, especially glucose, fructose, and lactose [21]. The AgNPs obtained with this innovative and entirely green methodology have more promising characteristics than those produced with chemical methods: the obtainable dimensions are a few tens of nanometers, the stability of the colloids is several months, and their antibacterial activity is preserved.

It is important to consider that the efficacy of a bioactive species depends not only on its intrinsic characteristics but also on how the substance is administered [22]. Therefore, it becomes crucial to develop new biomaterials capable of entrapping desired molecules, delivering them to the target site, and releasing them in a controlled way over time. In fact, a controlled and prolonged release is essential to ensure a safer and more effective dosage of drugs, preventing the body from being subjected to high drug concentrations that could lead to undesired side effects [23]. Hydrogels are promising biomaterials as controlled release systems. They consist of a water-swollen three-dimensional lattice in which it is possible to entrap a wide variety of bioactive substances, including AgNPs [24]. An interesting class of hydrogels consists of those based on peptides [3]. Self-assembling peptides are a particular class of molecules characterized by the ability to spontaneously organize themselves into ordered and stable structures in conditions of thermodynamic equilibrium, thanks to the formation of non-covalent bonds (hydrogen bonds, ionic, hydrophobic, and Van der Waals interactions) among the side chains of the amino acids of which they are made [25,26,27]. These intra- and inter-molecular interactions allow the peptide to organize into ordered secondary (α-helix and β-sheet) and tertiary (fibers and fibrils) structures. The possibility to obtain stable hydrogels using low molecular weight peptides has emerged in recent years since short peptides with appropriate chemistry can act as hydrogenators in specific pH or temperature conditions; they self-assemble into supramolecular structures, such as nanofibers, which give rise to the three-dimensional lattice which constitutes the solid phase of the hydrogel. During this process, an extremely high amount of water (often more than 90% of the total hydrogel weight) is trapped inside the meshes of the 3D network [28].

In this paper, we worked on the preparation of peptide hydrogel composites containing AgNPs (hgel@AgNPs) using glucose as a green reducing agent. As far as the peptide components are concerned, we used Fmoc-Phe_3_, a short self-assembling peptide, that we synthesized with a biocatalytic reaction. We have previously reported the optimization and characterization of the native hydrogel [13,28,29]. In this work, we used this system as a platform for the preparation of composite materials with antibacterial properties. The novelty of this work lies in the development of different synthetic methodologies for one-pot preparation of hgel@AgNPs, where AgNP formation occurs in situ and simultaneously with the sol–gel transition. The first synthesis involved the use of sodium 3-mercapto-1-propanesulfonate (3MPS) as a stabilizing agent and NaBH_4_ as a reducing agent. Then, we moved on to a green approach, exploiting the reducing properties of monosaccharides such as glucose. We compared the effect of the preparation technique of composite hydrogels on the size, monodispersity, and stability of in situ synthesized AgNPs. All the prepared hgel@AgNPs samples were characterized with UV-Vis spectrophotometry, Dynamic Light scattering (DLS) analysis, Scanning Electron Microscopy (SEM), and rheological analysis. Finally, composite hydrogels were tested against *Staphylococcus aureus* to evaluate their antimicrobial activity.

## 2. Materials and Methods

### 2.1. Materials

Fluorenylmethyloxycarbonyl-phenylalanine (FmocPhe, >99%) and diphenylalanine (Phe_2_, >99%) were from Bachem GmbH (Weil am Rhein, Germany) and used as received. Lipase from *Pseudomonas fluorescens* (PFL, 20.000 U/mg), silver nitrate (AgNO_3_, 98%), sodium 3-mercapto-1-propanesulfonate (3MPS), sodium borohydride (NaBH_4_), sodium dodecyl sulfate (SDS), β-D-glucose, and all other chemicals and solvents were from Sigma Aldrich (St. Louis, MO, USA) and used as received.

### 2.2. Hydrogel Biosynthesis

Fmoc-Phe_3_ tripeptide hydrogels were prepared starting from equimolar quantities of Fmoc-Phe and Phe_2_ according to previously reported procedures [30]. Fmoc-Phe_3_ is biosynthesized in an aqueous solution by PFL after 30 min of incubation at a controlled temperature. The reaction yield for Fmoc-Phe_3_ formation was calculated using an HPLC method, as described previously [30].

### 2.3. Synthesis of AgNPs in a Hydrogel Matrix

**Figure 1:**
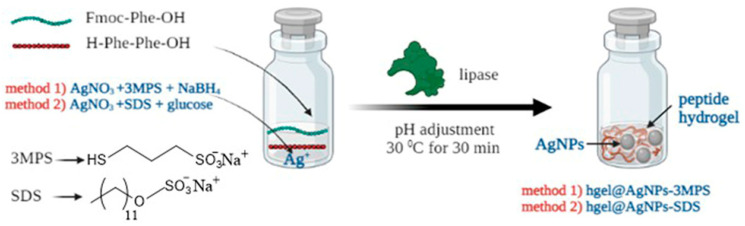
Scheme of the preparation of hgel@AgNPs composites.

AgNPs entrapped within the hydrogel matrix were synthesized using two bottom-up synthetic techniques: a more traditional one requiring NaBH_4_ as a reducing agent and a green one, in which a monosaccharide (β-D-glucose) is employed (Figure 1). In both cases, a hydrogel precursor solution and a AgNP precursor solution were prepared separately and then mixed. After adjusting pH to neutrality by adding 0.1 M HCl, the enzyme solution was added to start gelation, which occurred after incubation at 30 °C for 30 min.

When using NaBH_4_, the AgNP precursor solution was prepared as follows: 333 μL of AgNO_3_ (5 mM) and 333 μL of 3MPS (20 mM) were mixed and bubbled with Ar for 10 min. At the end of bubbling, 333 μL of NaBH_4_ (25 mM) were added to start the AgNPs nucleation. Regarding the synthesis using β-D-glucose, the AgNP precursor solution was prepared with hydrogel precursors and dissolved in an aqueous phase with the following reagents: 333 μL of AgNO_3_ (5 mM), 333 μL of SDS (10 mM), and 333 μL of β-D-glucose (75 mM) [31,32,33,34]. The optimized molar ratios among the reagents are equal to 1:2:15 (AgNO_3_:SDS:β-D-glucose). The mixture was then stirred magnetically for 1 min to start the nucleation of AgNPs.

### 2.4. UV-Vis Analysis and DLS Measurements

UV-Vis analyses were performed using a UV/Visibile Ultraspec 4000 spectrophotometer (Pharmacia Biotech, Uppsala, Sweden). DLS analyses were performed with a Zetasizer Nano ZS (Malvern Instruments, Malvern, UK). For DLS measurements, hydrogel samples were mechanically broken to obtain a liquid suspension and immediately diluted with deionized water to avoid NPs aggregation. All measurements were performed at room temperature and at least three times, calculating the average value ± standard deviation. Peak intensity analysis was used to determine the average hydrodynamic diameter of the scattering particles.

### 2.5. Inductively Coupled Plasma-Atomic Emission Spectrometry

The yield of AgNPs in situ formation within the hydrogels was calculated through Inductively Coupled Plasma Atomic Emission Spectrometry (ICP-AES) experiments. Hydrogel samples were prepared as described in Section 2.3 and mechanically broken to obtain a liquid suspension. The suspension was filtered through a 3 kDa Amicon Ultra-100 Centrifugal Filter Unit (Burlington, MA, USA) inside a centrifuge operating at 15,000× *g* rpm for 30 min at 20 °C in order to separate AgNPs from unreacted silver ions. The separated AgNPs were digested in HNO_3_. Ag concentration in the digestate was analyzed using a Varian Vista RL CCD Simultaneous ICP-AES spectrometer (338.289 nm Ag emission line was taken into account). The AgNPs formation yield was calculated with Equation (1):

yield% = (experimental Ag mass)/(theoretical Ag mass) × 100(1)

### 2.6. SEM Measurements

AgNPs morphology was studied with Scanning Electron Microscopy at the CNIS laboratory of Sapienza University. A drop of each sample was deposited on aluminum stabs and air-dried. Analyses were carried out using a Zeiss Auriga 405 microscope (Oberkochen, Germany) at a low extracting voltage and current.

### 2.7. Rheological Measurements

An Anton Paar MCR 302 rotational rheometer was used to record the elastic (G′) and viscous (G″) moduli of hydrogel samples. The instrument, equipped with a temperature control unit, used a plate-plate geometry. The gelation process was followed at 30 °C, applying a deformation of 1% and a constant frequency of 1 Hz in time sweep experiments. The elastic and viscous moduli of preformed hydrogels were measured in frequency sweep experiments, varying the applied frequency between 0.01 and 20 Hz at 30 °C.

### 2.8. Evaluation of Hydrogel Stability in Physiological Conditions

The degradation kinetics of the native hydrogels as well as hydrogel composites containing AgNPs, under physiological conditions, were evaluated using the following stability assay. Briefly, 8.5 mL of Ringer’s solution, containing NaCl (8.6 mg/mL), KCl (0.3 mg/mL), and CaCl_2_ (0.33 mg/mL), were added to each hydrogel sample. Samples were incubated at 37 °C for 30 days. After incubation, the supernatant was removed. The hydrogels were weighed before adding Ringer’s solution (W_0_) and after its removal (W_t_). The percentage of weight loss (ΔW%) was calculated using Equation (2):

ΔW% = (W_0_ − W_t_)/W_t_ × 100 (2)

To obtain the daily degradation rate (T), the percentage of weight loss (ΔW%) was then divided by the total incubation time (30 days) using Equation (3):

T = (ΔW%)/30(3)

### 2.9. Antibacterial Activity Studies

The antimicrobial activity of hgel@AgNPs composites was evaluated using the *S. aureus* strain from the American Type Culture Collection (ATCC) 25923. MICs (minimum inhibitory concentrations) were calculated using the broth microdilution method. The laboratory strain *S. aureus* ATCC 25923 was collected from a Blood Agar plate (Oxoid, Basingstoke, Hampshire, UK) and inoculated in 2 mL of 0.45% saline solution (Air Life, Fresno, CA, USA) to obtain turbidity of 0.5 ± 0.1 McFarland (McF). Subsequently, 100 µL of bacterial suspension, diluted 1:100 in cation-adjusted Mueller–Hinton broth (MHB), corresponding to 1 × 10^6^ CFU/mL, was used to inoculate a 96-well polystyrene plate (Corning Inc., Corning, NY, USA). The bacteria were incubated at 37 °C for 24 h in the presence of different concentrations of antimicrobial compounds.

After treatment with the different antimicrobial compounds, viable cells were determined with plate counting for the CFU/mL determination [34]. All experiments were in triplicate and repeated three times.

## 3. Results and Discussion

### 3.1. Synthesis of hgel@AgNPs Composites

Our research group has extensively studied the lipase-mediated biosynthesis of self-assembling peptide hydrogels [29,35]. Such systems can be prepared in mild reaction conditions and are highly versatile, allowing the incorporation of bioactive molecules, nanoparticles, or other molecules, serving as a tailorable platform for the preparation of composites with desired features for a wide variety of applications [13]. In this work, starting from our previously optimized protocols, we attempted to prepare antibacterial hydrogel composites containing AgNPs. We developed two different synthetic protocols, both based on the reduction of AgNO_3_, as described in Section 2.3: one was based on the use of chemical reducing agents such as NaBH_4_, while the other relied on environmentally friendly natural reducing agents such as β-D-glucose. It was necessary to optimize the amounts of reducing agents for in situ AgNP formation. Both synthetic protocols successfully yielded self-supporting hydrogels after approximately 30 min of incubation at 37 °C, with a tripeptide reaction yield of about 30%. Comparing these results with our previously published data on the native hydrogel, we can observe that AgNP precursors did not interfere with tripeptide formation and self-assembling [30].

### 3.2. UV-Vis Analysis

**Figure 2:**
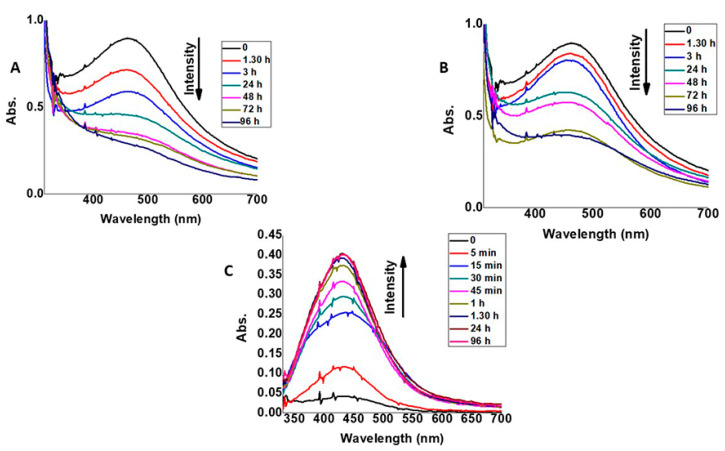
UV-Vis analysis in water. (**A**,**B**) The absorbance of hgel@AgNPs-3MPS at RT and 4 °C, respectively. (**C**) The absorbance of hgel@AgNPs-SDS at RT.

Aiming to characterize in situ formed AgNPs, their optical features such as the presence of SPR (surface plasmon resonance) band as a function of time were studied. Different experiments were performed to investigate the influence of storage temperature and duration on AgNP surface plasmon resonance values. Firstly, the evolution of AgNPs absorbance in the presence of NaBH_4_ as a function of reaction time was investigated. Samples stored at room temperature (RT) and 4 °C were examined. Figure 2A,B show the time evolution of the adsorption spectra of AgNPs synthesized in the hydrogel with NaBH_4_ and 3MPS at different temperatures. For both temperatures, the SPR band of AgNPs, centered at 475 nm, tends to decrease, and the plasmonic peak tends to widen over time. This is most likely due to the poor stability of AgNPs, which tend to aggregate. The reported data show that sample storage at 4 °C partially prevents aggregation, probably due to a reduction in diffusion phenomena within the hydrogel matrix.

As can be seen from the spectra (Figure 2C), the SPR band of AgNPs-SDS synthesized with β-D-glucose at RT increases over time, while in the case of NaBH_4_-mediated AgNPs synthesis, the SPR band decreases with time. In addition, for AgNPs synthesized with glucose, the SPR band is present at a smaller wavelength (425 nm), and it is narrower than the SPR peak of AgNPs synthesized with NaBH_4_. These experimental observations could be due to the different AgNP formation kinetics obtained with the two different reducing agents. NaBH_4_ is a strong reducing agent, used in combination with 3MPS thiol. AgNPs could be subjected to an oxidation process with the formation of an Ag_2_O shell [36]. As a result, aggregation equilibria occur, and the band gradually widens and decreases over time.

However, the use of a milder reducing agent such as β-D-glucose may account for a slower AgNP formation that increases with time. In addition, the presence of SDS as the capping agent seems to prevent AgNPs aggregation, resulting in a smaller and narrower SPR band.

### 3.3. Inductively Coupled Plasma-Atomic Emission Spectrometry

The yield of AgNPs formed from Ag^+^ ions, using both β-D-glucose and NaBH_4_ as reducing agents, was calculated on the basis of ICP-AES experiments. According to ICP data, the amount of formed AgNPs in the presence of β-D-glucose and NaBH_4_ is equal to 1.2 ± 0.14 and 4.2 ± 0.11 ppm, respectively. Dividing these amounts by the theoretical value indicates that the reaction yields are 23.5% and 82.3% for AgNPs formed using β-D-glucose and NaBH_4_, respectively. As expected, in the optimized reaction conditions, the use of NaBH_4_ leads to higher yields of formed AgNPs, given that this compound is a strong reducing agent.

### 3.4. DLS Measurements

**Figure 3:**
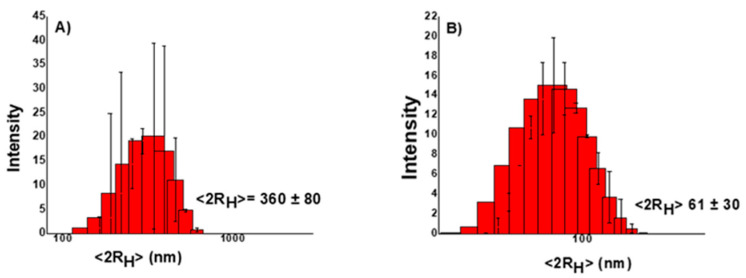
Hydrodynamic distribution of (**A**) hgel@AgNPs-3MPS and (**B**) hgel@AgNPs-SDS.

DLS measurements were performed to estimate AgNP size and polydispersion degree. The size of the AgNPs is a fundamental parameter to be considered in the realization of antimicrobial nanosystems. In fact, many of their chemical and physical properties, such as stability, reactivity (proportional to their surface/volume ratio), and release kinetics of Ag^+^ ions could be affected by size. The interaction processes of AgNPs with biological systems are also size dependent. Figure 3A,B shows the distributions of the hydrodynamic diameters of AgNPs synthesized using NaBH_4_ and β-D-glucose as reducing agents.

As can be seen, AgNPs synthesized using NaBH_4_ as a reducing agent have larger dimensions and polydispersion (~360 nm, PDI: 0.475), compared to those obtained using β-D-glucose (~60 nm, PDI: 0.233). Certainly, different factors may be responsible for such differences in AgNP dimensions and polydispersion. Higher AgNP concentrations may enhance particle aggregation within the hydrogel structure (as when using NaBH_4_). In addition, the structure of the capping agent could affect the final dimensions and/or particle aggregation.

### 3.5. SEM Measurements

**Figure 4:**
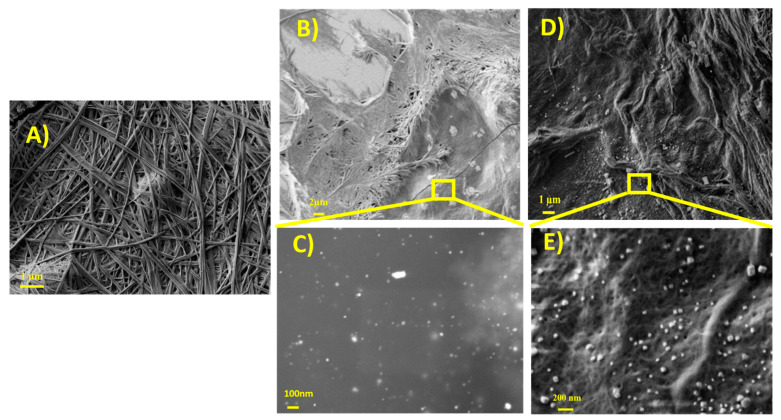
SEM images of (**A**) native hydrogel; (**B**) hgel@AgNPs-SDS; (**C**) hgel@AgNPs-SDS at high magnification; (**D**) hgel@AgNPs-3MPS; and (**E**) hgel@AgNPs-3MPS at high magnification.

The morphological features of the native hydrogel and hgel@AgNPs were investigated using SEM. Figure 4A shows a micrograph of the native hydrogel in which the fibrous nature of the highly reticulated three-dimensional network can be seen. Figure 4B–E is related to hgel@AgNPs-SDS and hgel@AgNPs-3MPS, respectively, and their high-resolution images. It can be seen that the presence of AgNPs does not affect the ability of the Fmoc-Phe3 tripeptide to self-assemble into supramolecular structures giving rise to composite gels with a highly cross-linked fibrillar morphology. Moreover, in both composites, AgNPs appear as well dispersed inside the hydrogel matrix.

### 3.6. Rheological Studies

**Figure 5:**
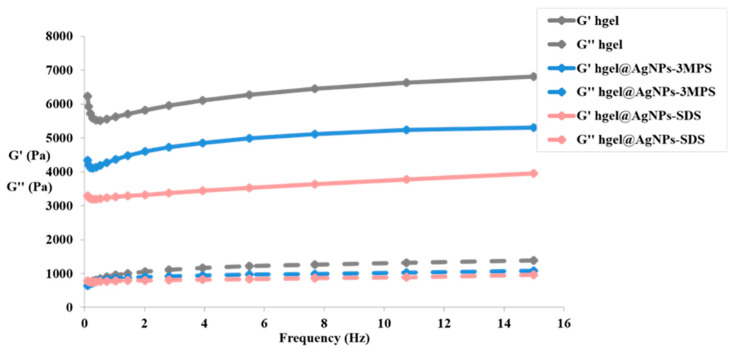
Frequency sweep of the hydrogel; hgel@AgNPs-3MPS; and hgel@AgNPs-SDS.

Studying the rheological features of hydrogels is pivotal for evaluating their application potential. With the aim to analyze the viscoelastic behavior of hydrogel composites, we used a dynamo-mechanical analysis, and the measurements were conducted in an oscillatory mode, at different application frequencies of the stimulus under constant strain. Both hydrogel samples containing AgNPs, formed in situ with the two different protocols described in Section 2.3, as well as the native control hydrogel, were analyzed. The aim of this study was to quantify the influence of in situ formed AgNPs on the viscoelastic behavior of the composites, and therefore how the elastic and viscous moduli of the hydrogels changed. The results for all samples are reported in Figure 5.

Regarding all types of hydrogels, the elastic modulus G′ and the viscous one G” do not vary according to the oscillation frequency but remain almost constant for the entire frequency range analyzed. On this basis, a solid-like behavior is found (G′ > G″), whatever the frequency of the stimulus to which the sample is subjected. This indicates that there is a gel or a three-dimensional network in which chains, in this case, peptides, are held together thanks to physical and chemical crosslinks that prevent their sliding (typical of a liquid system) even at low frequencies. In fact, in the gel, there are only conformational variations in the peptide chains and translational motions of these chains are absent. Furthermore, from the frequency sweep experiments conducted, it is possible to obtain information also regarding the strength of the gel. Since the elastic modulus G′ is much higher than the viscous one G″ for all samples, it is possible to state that they are in the presence of strong and stable hydrogels.

In particular, with hgel@AgNPs-3MPS, we can observe a reduction in the mechanical properties compared to the hydrogel alone. In this case, the 3MPS stabilizer and NaBH_4_ reducing agent could negatively influence the hydrogel gelling ability. In fact, physical crosslinks are present in the peptide hydrogel, such as hydrophobic interactions and aromatic π–π stacking (due to the presence of benzyl groups in the side chain of the amino acid phenylalanine), which can be reduced in the presence of polar compounds such as 3MPS and NaBH_4_. As far as the hgel@AgNPs-SDS samples are concerned, we obtained a significant reduction in the viscous modulus G″ and, in particular, of the elastic one (G′). This indicates that a weaker gel is present, probably due to the presence of the amphiphilic surfactant SDS. In fact, the latter has a polar head that could affect the hydrophobic interactions that hold together the peptide lattice of the hydrogel.

### 3.7. Study of Hydrogel Stability in Physiological Conditions

The stability of hydrogel composites in physiological conditions, being an important feature for biological applications, was evaluated. The obtained results are summarized in Table 1.

Overall, all samples demonstrated good stability, accounting for a weight loss of approximately 10% after 1 month. The reported values of weight loss percentage (ΔW%) and degradation rate (T) of the analyzed composites do not differ much from those obtained for the native hydrogel. Therefore, it is possible to state that the in situ synthesis of AgNPs, both through the use of NaBH_4_ and glucose, does not significantly compromise the stability of the peptide hydrogel in physiological conditions.

### 3.8. Antibacterial Properties of AgNPs and hgel@AgNPs Composites

To test the antibacterial effects of silver and the application of these nanocomposites in the biomedical field, it was decided to carry out in vitro inhibition tests against *S. aureus*, which is a major human pathogen and the most common Gram-positive bacteria isolated from skin ulcers [36].

The tests were carried out on the hgel@AgNPs composites made with the three different synthesis strategies and with a Ag concentration equal to 0.094 mg/mL. In addition, the three types of AgNPs in solution with [Ag] = 0.094 mg/mL and the hydrogel as it is in the absence of AgNPs were tested as controls.

A viability test was then carried out on *S. aureus* in the presence of these biomaterials at different concentrations to determine the MIC (minimum inhibitory concentration). It was evidenced that the hydrogel exhibited limited antibacterial activity even in the absence of AgNPs. Indeed, it is known that the peptides of which it is made up have antimicrobial properties [13]. In particular, the hydrogel was effective against *S. aureus* at the following concentrations: Fmoc-Phe: 0.9 mg/mL, Phe_2_: 0.725 mg/mL, and Fmoc-Phe_3_: 0.7 mg/mL. The results of the microbiological analyzes obtained for the AgNPs in solution and for the hgel@AgNPs composites prepared under the different experimental conditions are reported in Table 2.

Comparing the MIC data, it can be seen that there are no substantial differences either with the variation in the synthesis technique of AgNPs (using NaBH_4_ or β-D-glucose) or as a function of sample type (AgNPs colloidal suspension or hgel@AgNPs composites). In each of the analyzed cases, the MIC ranged between 0.004 and 0.008 mg/mL. Such values are comparable with those reported in the literature for similar systems. Thus, all the systems studied are promising candidates for treating *S. aureus* infections [13]. In particular, hgel@AgNPs samples have the advantage of being able to release silver ions in a controlled way over time; therefore, the bacteria, in the presence of these composites, will be subjected to the antimicrobial agent for a prolonged time and the duration of the antibacterial effect will be greater. Moreover, the green synthesis we propose, which uses glucose as a reducing agent, avoids the use of chemicals that could represent a biological risk.

## 4. Conclusions

In this study, peptide hydrogel composites containing AgNPs were synthesized. The AgNps were obtained with an in situ reduction of Ag^+^ using both traditional chemical reducing agents such as NaBH_4_ as well as natural reducers such as β-D-glucose. The use of β-D-glucose yielded composites containing AgNPs with improved physical features, e.g., smaller dimensions and greater stability over time, while allowing a more sustainable synthesis. The AgNPs synthesized in situ did not affect either the highly cross-linked fibrillar morphology of the hydrogel or its stability.

From the MIC data collected with the microbiological analyses, it appears that, in each of the experimental conditions used in the synthesis of AgNPs, the composites exhibit antibacterial activity against *S. aureus*. Therefore, it would be interesting to evaluate the bactericidal activity of these composites against other pathogenic bacteria such as the Gram-negative *Pseudomonas aeruginosa*.

Overall, the composite material prepared using β-D-glucose exhibits promising features and can easily be obtained using a sustainable approach. Therefore, such a composite will be further investigated to analyze the release kinetics of silver ions from the composite, with the prospect of its application in the biomedical field.

## Figures and Tables

**Table 1:** Percentage of weight loss (ΔW %) after 30 days of incubation in Ringer solution and daily degradation rate (T) of native and composite hydrogels.

Sample	ΔW %	T
Native hydrogel	9.11 ± 2.34	0.31 ± 0.08
hgel@AgNPs-SDS	10.89 ± 1.76	0.36 ± 0.06
hgel@AgNPs-3MPS	12.60 ± 2.65	0.49 ± 0.09

**Table 2:** Minimum inhibitory concentration (MIC) for *S. aureus* ATCC 25923 for AgNPs in solution and hgel@AgNPs samples.

Sample	Reducing Agent	MIC (mg/mL)
AgNPs-3MPS	NaBH4	0.008
hgel@AgNPs-3MPS	NaBH4	0.006
AgNPs-SDS	β-D-glucose	0.004
hgel@AgNPs-SDS	β-D-glucose	0.006

## Data Availability

Data are contained within the article.
